# Prevalence and S gene characterization of porcine epidemic diarrhea virus in Sichuan province, China (2023–2024)

**DOI:** 10.3389/fvets.2025.1748998

**Published:** 2026-01-26

**Authors:** Lu Xiao, Run-min Kang, Xue-jing Wu, Ji-feng Yu, Jun-yan Yang, Cong-jian Mao, Jing Xie, Yong-gang Ye, Xing-yu Li, Yong Wei, Zi-xuan Zeng, Wei-li Kang, Meng Pan, Ye Cao, Jian-qiang Ye, Yin Wang

**Affiliations:** 1 College of Veterinary Medicine, Sichuan Agricultural University, Chengdu, China; 2 Animal Genetic Breeding and Reproduction Key Laboratory of Sichuan Province, Sichuan Animal Science Academy, Chengdu, China

**Keywords:** G2b, molecular characterization, PEDV, prevalence, S gene

## Abstract

Porcine epidemic diarrhea virus (PEDV) is a major cause of devastating economic losses in the global swine industry. Sichuan Province, a major swine-producing region in China, is therefore a critical area for monitoring PEDV prevalence and evolution. We analyzed clinical samples collected from 365 diarrheic piglets across 70 pig farms located in 20 regions of Sichuan Province, China, from 2023 to 2024. The overall PEDV positivity rate was 40.27% (147/365). Prevalence varied considerably among regions, ranging from 0 to 63.64%, with over half (16/20) exceeding a 30% positivity rate, indicating widespread but heterogeneous circulation. Phylogenetic analyses based on the S gene from 33 representative strains revealed that they clustered into the G2b, G1c, and G2a subtypes, and these 33 PEDV S genes exhibited 94.3–99.9% nucleotide and 93.2–99.8% amino acid homology. The prevalent strains harbored frequent mutations in key antigenic sites of the S gene, including S10, SS6, and the collagenase equivalent (COE) domain. This study provides novel insights into the current epidemiology and genetic evolution of PEDV in China, which will inform more effective prevention and control strategies. Therefore, the control of the predominant G2b subtype should not be overlooked.

## Introduction

Porcine epidemic diarrhea (PED) is caused by porcine epidemic diarrhea virus (PEDV), a member of the genus *Alphacoronavirus*, family *Coronaviridae* (1, 2). This disease is characterized by severe symptoms, including vomiting, acute watery diarrhea, dehydration, and damage to the digestive tract mucosa, and it results in high mortality, particularly in neonatal piglets (3). PEDV infects pigs of all ages, and it is especially lethal to neonatal suckling piglets under 7 days old, while causing reduced production performance in adults. Infection with virulent PEDV strains can lead to up to 100% mortality (4–6). PED can be endemic or pandemic, depending on the viral strain responsible for the outbreak and the immune status of the pig population (7).

PEDV is an alphacoronavirus with a 28-kbp positive-sense single-stranded RNA (ssRNA) genome that contains at least seven open reading frames (ORFs) (8). The PEDV virion comprises a lipoprotein envelope and a nucleocapsid (9). The lipoprotein envelope consists of the S protein (spike protein), M protein (membrane protein), and E protein (envelope protein), all of which are situated outside the nucleocapsid. The nucleocapsid contains the N protein (nucleocapsid protein) and the viral genomic RNA (10). S protein, essential for viral attachment to host cells, is a type I transmembrane glycoprotein that forms a trimer on the viral surface, and it is composed of two subunits: S1 (aa 1–789) and S2 (aa 790–1,383) (11). The S1 protein contains three neutralizing epitopes: the collagenase equivalence (COE) region, epitope S10, SS2 and SS6 (12–14). The S gene is highly variable, amino acid substitutions, deletions, or insertions can significantly alter the virus’s pathogenicity and antigenicity (6, 15). The S1 domain plays a key role in modulating PEDV virulence *in vivo* and facilitating its growth adaptation *in vitro* (16, 17). According to S gene homology, PEDV strains are divided into classical (G1) and variant (G2) genotypes, however, there is no consensus on a more specific classification system. Initially, scholars referred to the strains that appeared before 2010, represented by CV777, as classical strains of PEDV (G1), and those discovered after 2010 as mutant strains (G2) (18). It has also been reported that PEDV has been classified into six subtypes: G1a, G1b, G1c, G2a, G2b, and G2c, based on the homology of the S gene (19, 20). It was also reported that PEDV is divided into G1a, G1b, G2a, G2b, and G2c (21–23). The S gene is highly variable. It plays a crucial role in elucidating the genetic relationships among PEDV field strains, tracking viral epidemiology, and informing vaccine development.

Sichuan Province is one of China’s largest swine-producing regions, consistently leading the nation in annual slaughter volume. As a strategic swine production hub in central-western China, it reported slaughter volumes of 66.627 million and 61.496 million heads in 2023 and 2024, respectively (National Bureau of Statistics and Sichuan Survey Office), representing 9.2 and 8.8% of the national total. Consequently, the PEDV epidemic profile in Sichuan holds significant indicative value for informing national-level prevention and control measures. Therefore, this study was aimed to elucidate the epidemiological dynamics of PEDV, a key threat to the swine industry. A total of 365 diarrheal samples were collected from 70 pig farms across multiple cities in Sichuan between October 2023 and December 2024. These samples were screened for PEDV using quantitative RT-PCR (qRT-PCR), the S gene from representative positive samples was subjected to genetic evolutionary analysis. The resulting molecular characterization of circulating PEDV strains informs our understanding of viral evolution and provides critical data for developing targeted interventions.

## Materials and methods

### Ethic statement

All animal experiments in this study were conducted in accordance with the guidelines and under the approval of the Sichuan Animal Science Academy (Approval No. 2023022). No research-induced sacrifice or euthanasia occurred. Compliance with Chinese Regulations for Experimental Animals was confirmed.

### Clinical sample information

**Figure 1:**
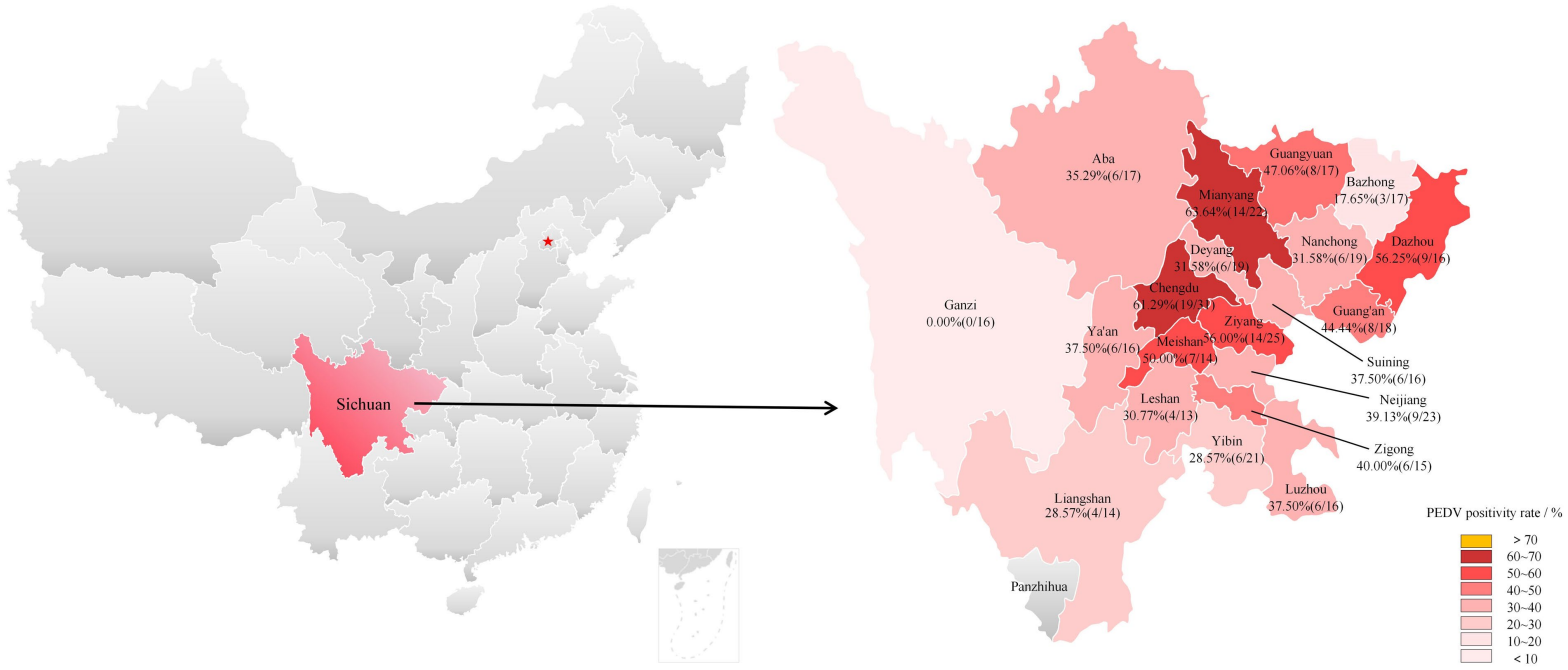
Locations of samples and PEDV detections in Sichuan. The locations marked with red five-pointed star signify Beijing, the capital city of China. Cities that did not participate in the sample collection are marked in gray. The PEDV positivity rate for each region is presented as percentage (number of positive samples/total number of samples).

Between October 2023 and December 2024, 365 samples were collected from diarrheic piglets on 70 farms across 20 major prefectures in Sichuan Province, China, covering 20 of the province’s total 21 prefectures (Figure 1). The map was based on the standard map (No. GS(2020)4398) from China’s Ministry of Natural Resources. To visually highlight sample distribution and positivity rates across Sichuan prefectures, the vector file was processed in Microsoft PowerPoint, where areas were color-coded and labeled with prefecture names and positivity rates. No original geographic boundaries were altered. The samples comprised two types: non-invasive fecal samples collected from live swine without restraint during routine farm management, and intestinal tissues obtained post-mortem from swine that died of natural diarrheal disease within 2 h. All samples were documented, and stored at −80 °C until analysis.

### RNA extraction and preparation of cDNA

Samples were transferred to sterile 1.5 mL centrifuge tubes in PBS, homogenized, and subjected to three freeze–thaw cycles. Total RNA was then extracted from diarrheal stools or intestinal samples using the FineMag Rapid magnetic bead Method Virus DNA/RNA Extraction Kit (GENFINE, China), following the manufacturer’s instructions. The cDNA was synthesized using the All-In-One 5 × RT MasterMix Kit (Applied Biological Materials Inc., Canada), also following the manufacturer’s instructions. The cDNA was stored at −80 °C, prepared for further experimental analysis.

### Real-time PCR detection

Total RNA was extracted from the clinical samples and reverse-transcribed into cDNA. For PEDV detection, a SYBR Green-based quantitative real-time PCR (qRT-PCR) assay was employed. Specific primers were targeting a relatively conserved region of the PEDV M gene (GenBank: MZ712170.1) were designed using DNASTAR and Oligo software, yielding an amplicon of 103 bp. The primer sequences were as follows: PEDV-M-F: 5’-TTCTATTCCCGTCGATGAGGTGAT-3′ and PEDV-M-R: 5’-GACACCATACAAGAACGCAGA-3′. The qPCR assay was conducted in a 25 μL solution consisting of 12.5 μL of 2 × TB Green Premix Ex Taq II (Tli RNaseH Plus; Takara, Japan), 1 μL of 10 mM PEDV-M-F/R, 2 μL of cDNA as the template, and 8.5 μL of ddH_2_O. The amplification was conducted as follows: 95 °C for 30 s, 95 °C for 5 s, 60 °C for 30 s (40 cycles), followed by 95 °C for 5 s, 60 °C for 1 min, and 95 °C for 15 s. A sample was considered positive if the initial *Ct* value was <35. Samples with *Ct* values ≥35 but <40 were retested, and only samples with a confirmed *Ct* ≥35 or with undetermined results (no amplification) were defined as negative.

### PEDV S gene sequencing

Four sets of amplification primers were used to amplify the S gene (24). To ensure sequence accuracy, amplifications were performed using 2 × PrimeSTAR LongSeq Premix (Takara, Japan), a high-fidelity DNA polymerase mix. The protocol was carried out in a 25 μL reaction using 12.5 μL of 2 × PrimeSTAR LongSeq Premix, 1 μL of 10 μM forward/reverse primer, 2 μL of cDNA, and 8.5 μL of ddH_2_O. The PCR was performed under the following conditions: 94 °C for 1 min, followed by 40 cycles of 98 °C for 10s, 55 °C for 15 s, 68 °C for 1 min, and 98 °C for 20s, 68 °C for 30s. To obtain sequences completely independent of the amplification primers and to ensure the integrity of the termini, the purified PCR products were cloned into the pMD19-T vector (Takara, Japan). The purified PCR fragments were subsequently inserted into the pMD19-T cloning vector through ligation. Expression vectors were introduced into DH5α competent cells (Takara, Japan) for propagation. The resulting constructs were subsequently submitted to Beijing Tsingke Biotech Co., Ltd. (Beijing, China) for sequencing.

### Sequence analyses

Representative strains were selected for full-length S gene sequencing through a tiered strategy: one sample per farm was chosen to avoid overrepresentation; genetically similar strains from adjacent areas were excluded; and samples with lower *Ct* values (<25) were prioritized to ensure amplification success. MegAlign (Lasergene package) was employed to align our experimental PEDV S gene sequences with GenBank reference sequences (Supplementary Table S1). Phylogenetic trees based on the S gene were built using the neighbor-joining method in MEGA 11 software. The robustness of the phylogenetic tree was evaluated by bootstrapping using 1,000 replicates. Heatmaps of nucleotide and amino acid sequence homology were generated using GraphPad Prism 10. The homology matrices were imported into the software and visualized using a “single gradient” color map, in which the color gradient represents the degree of homology.

## Results

### RT-qPCR survey of clinical samples

**Figure 2:**
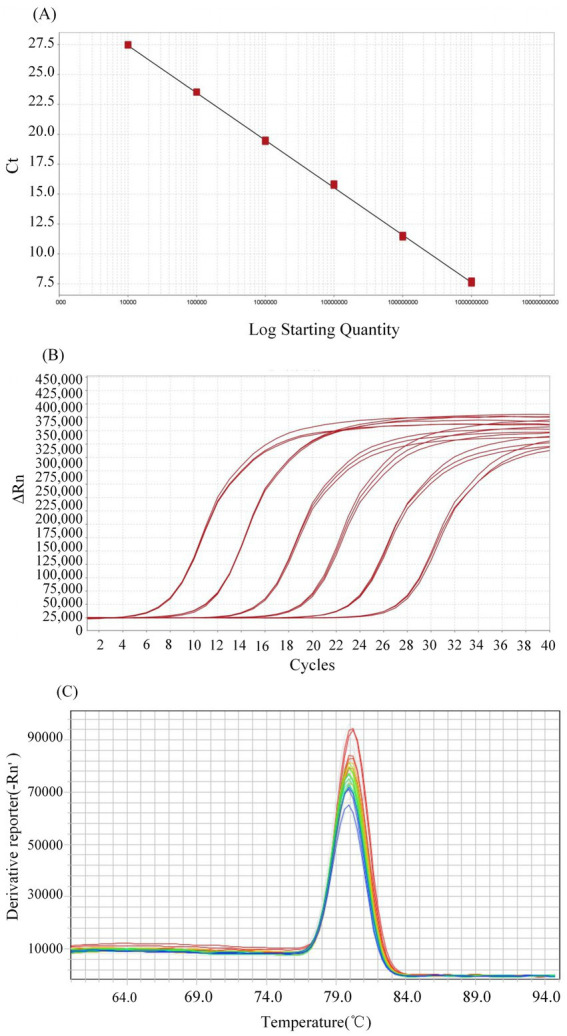
The RT-qPCR for PEDV detection. qPCR validation: **(A)** Standard curve (*y* = −3.885*x* + 42.677; efficiency: 90.9%, *R*^2^ = 0.998); **(B)** Amplification curves (consistent repeatability, linear dynamic range); **(C)** Melt curve (single peak, Tm ~ 80 °C).

Based on the need to investigate the prevalence and distribution of PEDV in Sichuan, 365 fecal swabs and small intestinal samples were collected from diarrheic pigs across 70 farms between October 2023 and December 2024. A specific RT-qPCR assay was developed (Figure 2) and applied for pathogen detection. Of the 365 samples tested, 147 were positive. Collectively, samples with *Ct* values between 20 and 30 accounted for the majority (90.5%, 133/147) of positive detections. PEDV circulation, with an overall sample-level positivity rate of 40.27% (147/365) and a farm-level infection rate of 68.57% (48/70). However, this high prevalence masked substantial geographical heterogeneity, as detection rates among the 20 surveyed prefecture-level divisions ranged from 0% (Ganzi) to 63.64% (Mianyang; Figure 1). Notably, the majority of regions (16/20, 80%) exhibited positivity rates exceeding 30%, with central and eastern prefectures such as Chengdu (61.29%) and Ziyang (56.00%) identified as significant hotspots.

### Molecular characterization of PEDV S gene

**Figure 3:**
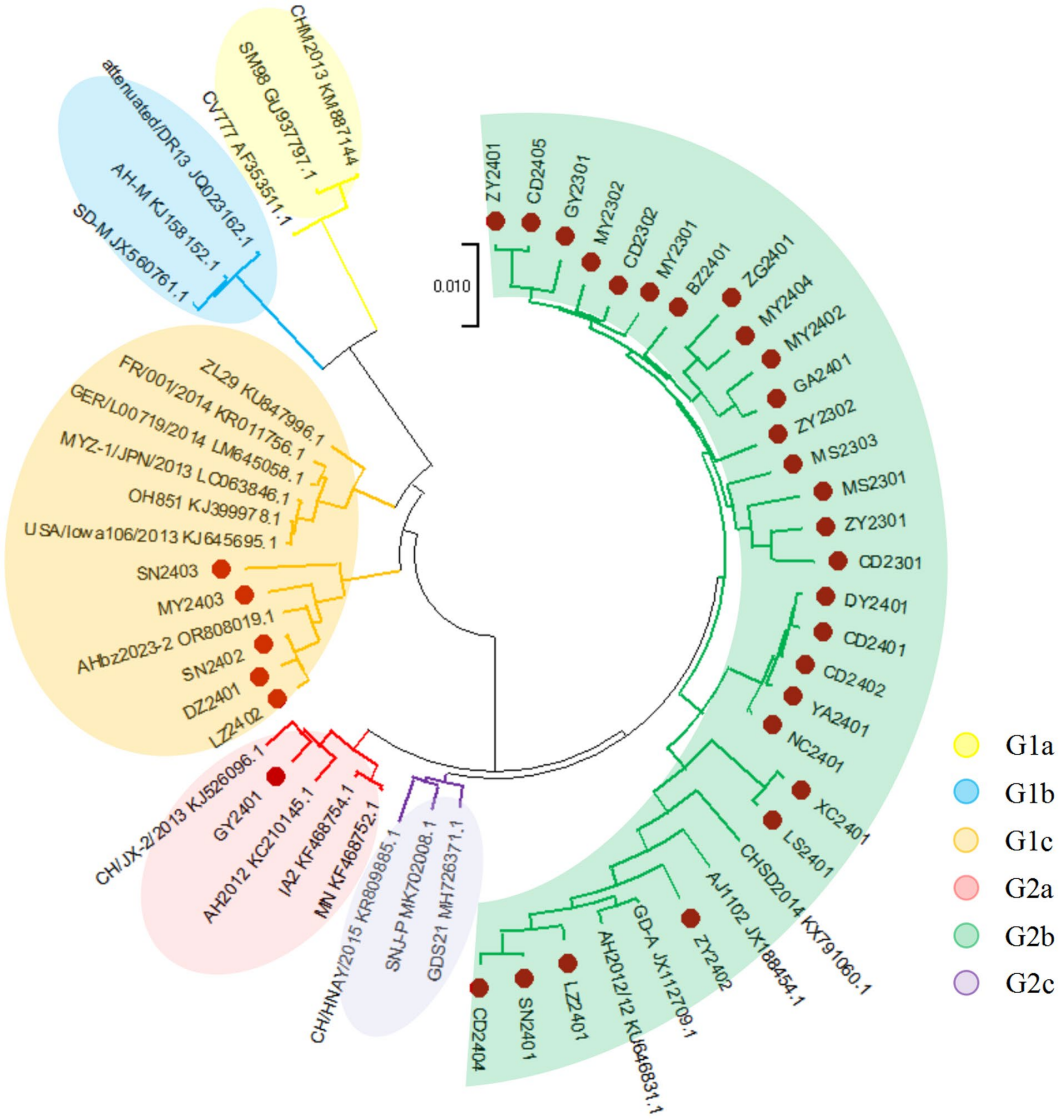
Phylogenetic analysis of PEDV strains based on S gene. Strains obtained in this study are labeled with solid red circles for differentiation. The phylogenetic tree was generated using MEGA 11’s neighbor-joining algorithm (1,000 bootstrap replicates).

**Figure 4:**
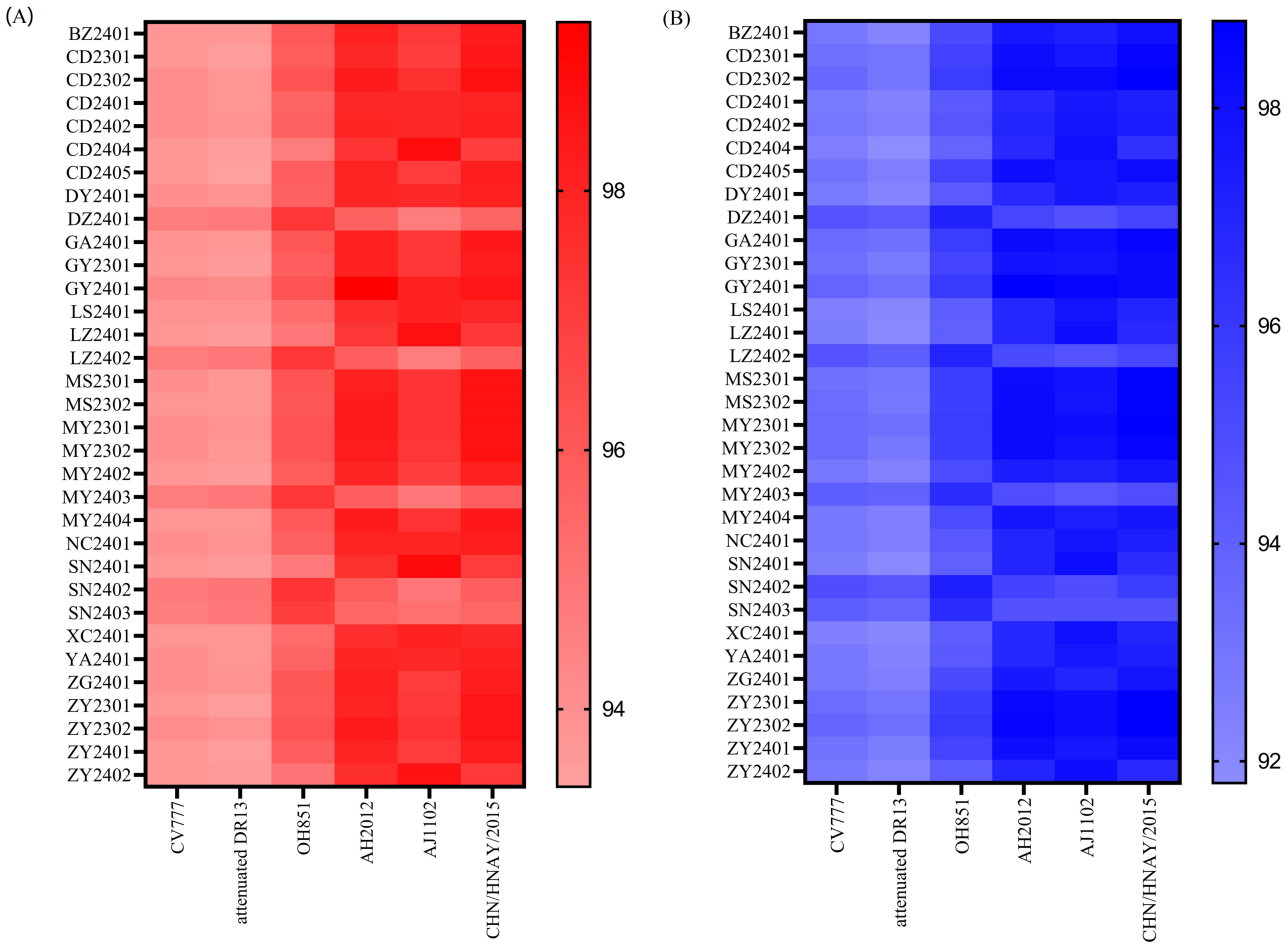
Heatmap showing homology analysis of the PEDV S gene. **(A)** Nucleotide homology among the study and reference strains; **(B)** Amino acid homology among the study and reference strains.

Following the selection strategy, representative strains were amplified for the full-length S gene by RT-PCR (Supplementary Figure S1). S gene sequencing of the 33 strains revealed lengths ranging from 4,148–4,161 bp, and distributed as follows: CD2402, LS2401, LZ2401, SN2401, XC2401, YA2401, and ZY2402 were each 4,157 bp; ZG2401, MY2404, and MY2402 were each 4,160 bp; DZ2401, LZ2402, and SN2402 were each 4,149 bp; MY2403 and SN2403 were each 4,148 bp; DY2401 and CD2401 were each 4,155 bp; and remaining strains exhibited S gene lengths of 4,161 bp. The results indicated that the nucleotide and amino acid sequences of the 33 strains collected in this study exhibited diversity and a high mutation rate. Among the 33 PEDV S genes, sequence identity ranged from 94.3 to 99.9% at the nucleotide level and 93.2 to 99.8% at the amino acid level (Supplementary Table S2). The 33 PEDV S gene sequences obtained in this experiment were grouped based on their alignment with reference sequences downloaded from GenBank. The sequences evolved into two distinct clades, designated G1 and G2. Each of these clades was further divided into three subclades, namely G1a, G1b, G1c, and G2a, G2b, G2c, respectively. A phylogenetic tree was then constructed using the neighbor-joining method with 1,000 bootstrap replications, facilitated by the MEGA 11 software. The phylogenetic analysis revealed that in our data, G2b, G1c, and G2a accounted for 81.8% (27/33), 15.2% (5/33), and 3.0% (1/33), respectively (Figure 3). 27 epidemic wild PEDV strains were located on the G2b branch, 5 belonged to the group G1c and only one belonged to the group G2a, which were all distantly related to the typical vaccine strains (CV777 and Attenuated DR13). Obviously, G2b strains were still the most prevalent in China, which were more similar to the strains of the original outbreaks in China (AJ1102, CHSD2014, GD-A, etc.). As for SN2403, MY2403, SN2402, DZ2402 and LZ2402 located on the G1c branch were closer to the AHbz2023-2 strain. GY2401 belongs to G2a was loser to the CH/JX-2/2013 strain. The nucleotide and amino acid homology of the S gene from the 33 strains collected in this study, compared with reference strains CV777 (G1a), attenuated DR13 (G1b), OH851 (G1c), AH2012 (G2a), AJ1102 (G2b), and CHN/HNAY/2015 (G2c), are visualized in the heatmaps shown in Figure 4. The complete homology data are provided in Supplementary Table S2.

### Comparative analysis of amino acid sequences of PEDV S protein

**Figure 5:**
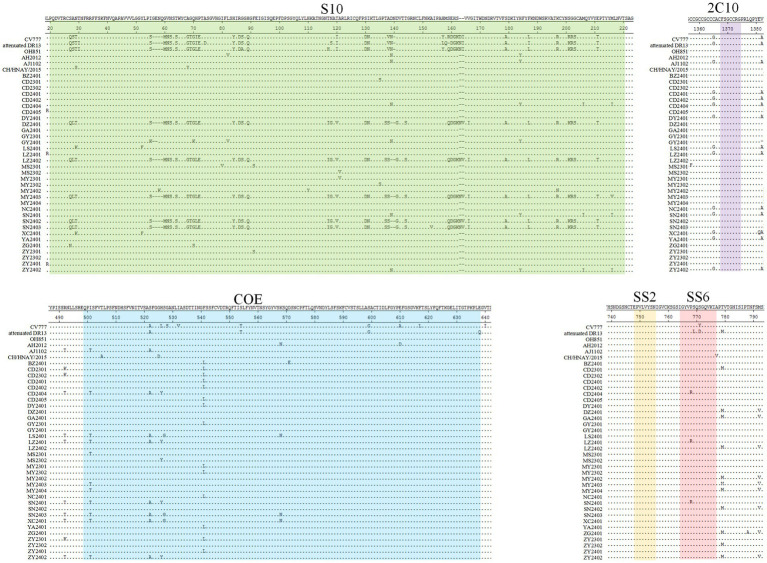
PEDV S protein amino acid alignment. The neutralizing epitopes S10 (green), COE (blue), SS2 (yellow), SS6 (red), and 2C10 (purple) are shown.

PEDV S protein epitopes were profiled, focusing specifically on five major neutralizing sites. These include those found in the vaccine strains CV777, attenuated DR13, OH851, AH2012, AJ1102, and CHN/HNAY/2015 (Figure 5). These epitopes comprise S10 (20–220 aa), COE (499–638 aa), SS2 (748–755 aa), SS6 (764–771 aa), and 2C10 (1368–1,374 aa) (25). Major amino acid variations were identified in the COE region and SS6 epitope. In contrast, the SS2 and 2C10 were relatively conserved, with no mutations detected.

Antigenic epitope S10 harbored 59 aa mutations across the 33 PEDV S proteins versus representative subtype strains. Crucially, G1 genotypes exhibited significant inter-group variation (DZ2401, LZ2402, MY2403, SN2402, SN2403, CV777, attenuated DR13 and OH851) and G2 genotypes (BZ2401, CD2301, CD2302, CD2401, CD2402, CD2404, CD2405, DY2401, GA2401, GY2301, GY2401, LS2401, LZ2401, MS2301, MS2302, MY2301, MY2302, MY2402, MY2404, NC2401, SN2401, XC2401, YA2401, ZG2401, ZY2301, ZY2302, ZY2401, ZY2402, AH2012, AJ1102 and CH/HNAY/2015) in this epitope, mutations included alterations such as 27QST30 or 27QSL30 to 27SAN30, 60MNSSS64 to 60GVNST64, 68GTGIE72 to 68AGQHP72, 84YIDSGQ89 to 84HIRGGH89, 130DN131 to 130SI131, 141DVTT144 to 141GVTS144, 159QDGK163 to 159SEHS163, 201KRS203 to 201SGG203. Other notable modifications were the deletion of four positions at 56GENQ59, two positions at 163NI164. There were also point mutations like N29K, Y53F, N58K, Q70S, Q70K, I80V, L82V, F91S, H110Y, A121V, G135S, A153V, S179A, H184Y, F187L, K197R, M206I, E211T, M216I. Eight aa substitutions were identified in the COE epitope, including I501T, S522A, H526Y, S527G, F541L, K568N, D571E. The SS6 epitope exhibited only one amino acid mutations, P768R. No mutations were observed in the SS2 and 2C10 epitope.

## Discussion

Porcine diarrheal disease poses a major threat to the pig industry in China. Among its numerous causative pathgens, PEDV remains the leading cause. Since its initial detection in China in the 1980s, PEDV has continually evolved, with highly pathogenic variant strains emerging around 2010 and triggering widespread national outbreaks (26). These variant strains are characterized by enhanced transmissibility and high mortality rates in suckling piglets, against which current vaccines based on classical strains provide limited protection (27, 28). PEDV continues to pose a significant threat to the swine industry in China, with Sichuan Province being a notably affected region.

Sichuan Province as a major swine-producing province in China, underscores the necessity of understanding the current prevalence and genetic characteristics of PEDV for developing effective control strategies. This study investigated PEDV in 20 prefecture-level cities across Sichuan, covering nearly all major swine-producing areas of the province from October 2023 to December 2024. The overall positivity rate was 40.27% (147/365). From these positive samples, 33 representative strains were selected for in-depth phylogenetic analysis, 27 of them classified them into G2b, 5 were G1c, and 1 was G2a subtype. Our findings exhibited certain discrepancies with recent reports from both within and outside Sichuan Province, collectively delineating a complex epidemiological landscape. Within Sichuan, earlier surveillance (2020–2022) across eight prefectures, encompassing both gateway and inland areas, revealed a co-circulation of G2c, G2a, G2b, and G1b strains, representing a transitional phase (29). In contrast, a subsequent focused study in 2025 on key provincial gateways and hubs for swine transportation (e.g., Mianyang, Guangyuan, Luzhou, Chengdu) identified G2c as the dominant genotype, likely reflecting input or focal outbreak strains introduced through these critical nodes (30). Meanwhile, surveillance in 2023–2024, which excluded peripheral regions and a major southern gateway, reported G2a (87.5%, 14/16) as the predominant subtype in the remaining extensive area, potentially indicating the persistence of a historical dominant strain within the relatively enclosed internal circulation network of the Sichuan basin (22). These disparities strongly suggest that PEDV prevalence within Sichuan is not homogeneous, even during overlapping periods. This discrepancy can be attributed to three interrelated factors: differences in sampling strategies across studies, the unique ecological internal circulation within the Sichuan Basin, and the inherent spatiotemporal heterogeneity of PEDV. First, differences in the geographical and husbandry niches covered by various sampling networks also lead to the detection of different dominant strains. For example, studies focusing on inter-provincial gateways are more likely to identify introduced strains (e.g., G2c), while broader regional surveillance may reflect strains circulating within internal networks that are potentially more adapted (e.g., G2a or G2b). Second, the relatively enclosed geographical environment and the dense, complex farming ecology within the basin create a unique regional virus circulation system. This facilitates the long-term adaptation and dominance of specific strains, such as G2b, within the region. Additionally, PEDV prevalence itself exhibits high spatiotemporal heterogeneity, with different subtypes alternately forming local dominance in various regions, periods, and farming systems. For instance, the G2a subtype remains absolutely dominant in regions such as Guangxi and Henan (21, 31). In contrast, G2b and recombinant PEDV subtypes have been identified as the unequivocal epidemic backbone in a five-province region of southern China from 2021 to 2023 (32). Another a long-term monitoring study in Yunnan (2013–2022) reported that the G2b strain accounted for as high as 85.71% of cases (33). Thus, the observed dominance of G2b in our study reflects this complex epidemic ecology within a specific spatiotemporal context. Therefore, effective control must move beyond a one-size-fits-all approach and be grounded in real-time local surveillance. This involves implementing a comprehensive control strategy centered on precisely matching vaccination programs to the locally circulating dominant strains identified through surveillance, enforcing strict biosecurity protocols across all key points to prevent virus introduction and internal spread, conducting active pathogen monitoring and early-warning surveillance in critical populations, and integrating optimized husbandry management with regional coordinated control to enhance overall resilience.

The S protein serves as the primary immunogen of PEDV, not only crucial for eliciting neutralizing antibodies but also fundamental for mediating viral entry into host cells (34, 35). The identified antigenic sites on the PEDV S protein encompass the S10, COE, and the short peptide regions SS2, SS6, and 2C10 (36, 37). In this study, we conducted a comparative analysis of the S protein amino acid sequences from the 33 detected strains with representative strains from each subgroup. The results indicated that G2 genotype strains share the same insertions (“G56ENQ59” and “N140”) and deletions (“D/N163,” “I164”) when compared to G1 genotype strains. Comparative analysis with major vaccine strains (CV777 and AJ1102) revealed that the five G1c strains possessed distinct amino acid profiles across key antigenic domains (S10, COE, SS6). This variation pattern aligns with natural field virus evolution rather than vaccine reversion. Therefore, these strains likely represent circulating wild-type viruses, either as persisting local lineages or external introductions, underscoring the genetic complexity of PEDV in Sichuan. Notably, the S10 epitope displayed elevated variability, while SS6 and COE exhibited moderate mutation rates. Conversely, SS2 and 2C10 epitopes remained fully conserved at the residue level. Unique mutations in PEDV S protein, distinct from reference strains, were identified in the study isolates. These mutations varied across different strains, reflecting the diversity and dynamic evolution within the PEDV population, particularly those affecting potential linear B-cell epitopes, may enable the virus to effectively evade the host’s immune response. This variation underscores the adaptability of PEDV to its host, potentially enabling the virus to evade the immunity provided by current vaccines. This makes the effective prevention and control of PEDV more complex and challenging, suggesting that the continued development and adaptation of vaccines remain highly necessary.

Limitations of this study should be acknowledged. First, the sampling timeframe (October 2023 to December 2024) was relatively short, and sample sizes in some regions remained limited, which may not fully capture long-term trends or the complete geographical distribution of the virus. Second, to ensure sequencing success and avoid overrepresentation of individual farms, only 33 strains were selected for sequencing from the positive samples. This strategy, while ensuring data quality and focusing on dominant epidemic strains, may somewhat underestimate the within-farm genetic diversity and might not fully include variants with very low viral loads. Nevertheless, all selection steps were based on clear biological and technical rationale, and the data presented still reliably reflect the predominant characteristics of PEDV strains currently circulating in Sichuan, providing an important foundation for further extensive monitoring and research.

## Conclusion

This surveillance in Sichuan from October 2023 to December 2024 revealed a PEDV positivity rate of 40.27% (147/365). Sequence analysis identified G2b as the dominant subtype within the region. The relatively enclosed geography of the Sichuan Basin may foster an internal circulation pattern that could contribute to the establishment of this local dominance. Prevalent mutations were identified in key antigenic domains (S10, COE, SS6). These findings confirm the active circulation and genetic diversity of PEDV in the region. Consequently, effective control strategies should be informed by real-time local surveillance to address such region-specific viral ecology through targeted interventions.

## Data Availability

The datasets presented in this study can be found in online repositories. The names of the repository/repositories and accession number(s) can be found in the article/Supplementary material.

## References

[1] Chen Y Liu Q Guo D. Emerging coronaviruses: genome structure, replication, and pathogenesis. J Med Virol. (2020) 92:2249. doi: 10.1002/jmv.26234, 32881013 PMC7435528

[2] Lin F Zhang H Li L Yang Y Zou X Chen J . PEDV: insights and advances into types, function, structure, and receptor recognition. Viruses. (2022) 14:1744. doi: 10.3390/v14081744, 36016366 PMC9416423

[3] Sun RQ Cai RJ Chen YQ Liang PS Chen DK Song CX. Outbreak of porcine epidemic diarrhea in suckling piglets, China. Emerg Infect Dis. (2012) 18:161–3. doi: 10.3201/eid1801.111259, 22261231 PMC3381683

[4] Jung K Saif LJ Wang Q. Porcine epidemic diarrhea virus (PEDV): An update on etiology, transmission, pathogenesis, and prevention and control. Virus Res. (2020) 286:198045. doi: 10.1016/j.virusres.2020.198045, 32502552 PMC7266596

[5] Luo H Liang Z Lin J Wang Y Liu Y Mei K . Research progress of porcine epidemic diarrhea virus S protein. Front Microbiol. (2024) 15:1396894. doi: 10.3389/fmicb.2024.1396894, 38873162 PMC11169810

[6] Stott CJ Temeeyasen G Tripipat T Kaewprommal P Tantituvanont A Piriyapongsa J . Evolutionary and epidemiological analyses based on spike genes of porcine epidemic diarrhea virus circulating in Thailand in 2008-2015. Infect Genet Evol. (2017) 50:70–6. doi: 10.1016/j.meegid.2017.02.014, 28235643 PMC7185621

[7] Wang X Chen B Yu R Si F Xie C Li Z . Magnolol, a Neolignan-like drug, inhibits porcine epidemic diarrhea virus replication in cultured cells. Pathogens. (2023) 12:263. doi: 10.3390/pathogens12020263, 36839535 PMC9965036

[8] Feng B Li C Qiu Y Qi W Qiu M Li J . Genomic characterizations of porcine epidemic diarrhea viruses (PEDV) in diarrheic piglets and clinically healthy adult pigs from 2019 to 2022 in China. Animals (Basel). (2023) 13:1562. doi: 10.3390/ani13091562, 37174599 PMC10177568

[9] Kirchdoerfer RN Bhandari M Martini O Sewall LM Bangaru S Yoon KJ . Structure and immune recognition of the porcine epidemic diarrhea virus spike protein. Structure. (2021) 29:385–392.e5. doi: 10.1016/j.str.2020.12.003, 33378641 PMC7962898

[10] Lee C. Porcine epidemic diarrhea virus: An emerging and re-emerging epizootic swine virus. Virol J. (2015) 12:193. doi: 10.1186/s12985-015-0421-2, 26689811 PMC4687282

[11] Wrapp D McLellan JS. The 3.1-angstrom cryo-electron microscopy structure of the porcine epidemic diarrhea virus spike protein in the prefusion conformation. J Virol. (2019) 93:e00923-00919. doi: 10.1128/jvi.00923-19, 31534041 PMC6854500

[12] Chiou HY Huang YL Deng MC Chang CY Jeng CR Tsai PS . Phylogenetic analysis of the spike (S) gene of the new variants of porcine epidemic diarrhoea virus in Taiwan. Transbound Emerg Dis. (2017) 64:157–66. doi: 10.1111/tbed.12357, 25903998

[13] Li F Zeng Y Zhang R Peng K Jiang C Xu Z . Genetic variations in S gene of porcine epidemic diarrhoea virus from 2018 in Sichuan Province, China. Vet Med Sci. (2020) 6:910–8. doi: 10.1002/vms3.326, 32885908 PMC7738707

[14] Sun YG Li R Xie S Qiao S Li Q Chen XX . Identification of a novel linear B-cell epitope within the collagenase equivalent domain of porcine epidemic diarrhea virus spike glycoprotein. Virus Res. (2019) 266:34–42. doi: 10.1016/j.virusres.2019.04.003, 30965063

[15] Park SJ Kim HK Song DS Moon HJ Park BK. Molecular characterization and phylogenetic analysis of porcine epidemic diarrhea virus (PEDV) field isolates in Korea. Arch Virol. (2011) 156:577–85. doi: 10.1007/s00705-010-0892-9, 21210162 PMC7086862

[16] Sato T Takeyama N Katsumata A Tuchiya K Kodama T Kusanagi K. Mutations in the spike gene of porcine epidemic diarrhea virus associated with growth adaptation in vitro and attenuation of virulence in vivo. Virus Genes. (2011) 43:72–8. doi: 10.1007/s11262-011-0617-5, 21559974 PMC7088782

[17] Zuo Q Zhao R Liu J Zhao Q Zhu L Zhang B . Epidemiology and phylogeny of spike gene of porcine epidemic diarrhea virus from Yunnan, China. Virus Res. (2018) 249:45–51. doi: 10.1016/j.virusres.2018.03.008, 29548744

[18] Huang YW Dickerman AW Piñeyro P Li L Fang L Kiehne R . Origin, evolution, and genotyping of emergent porcine epidemic diarrhea virus strains in the United States. MBio. (2013) 4:e00737-00713. doi: 10.1128/mBio.00737-13, 24129257 PMC3812708

[19] Han X Liu Y Wang Y Wang T Li N Hao F . Isolation and characterization of porcine epidemic diarrhea virus with a novel continuous mutation in the S1(0) domain. Front Microbiol. (2023) 14:1203893. doi: 10.3389/fmicb.2023.1203893, 37275149 PMC10232790

[20] Tian Y Yang X Li H Ma B Guan R Yang J . Molecular characterization of porcine epidemic diarrhea virus associated with outbreaks in Southwest China during 2014-2018. Transbound Emerg Dis. (2021) 68:3482–97. doi: 10.1111/tbed.13953, 33306274

[21] Shi K Li B Shi Y Feng S Yin Y Long F . Phylogenetic and evolutionary analysis of porcine epidemic diarrhea virus in Guangxi Province, China, during 2020 and 2024. Viruses. (2024) 16:1126. doi: 10.3390/v16071126, 39066288 PMC11281377

[22] Wu F Xu T Lai SY Ai YR Zhou YC Ge LP . Prevalence and genetic evolution analysis of porcine epidemic diarrhea virus and porcine circovirus type 2 in Sichuan Province, China, from 2023 to 2024. Front Vet Sci. (2024) 11:1475347. doi: 10.3389/fvets.2024.1475347, 39539315 PMC11558041

[23] Yao X Qiao WT Zhang YQ Lu WH Wang ZW Li HX . A new PEDV strain CH/HLJJS/2022 can challenge current detection methods and vaccines. Virol J. (2023) 20:13. doi: 10.1186/s12985-023-01961-z, 36670408 PMC9859669

[24] Luo J Song C Zhang T Li J Yang M Wang H. Isolation and characterization of porcine epidemic diarrhea virus with mutations in the spike gene in China. Virology. (2024) 600:110224. doi: 10.1016/j.virol.2024.110224 39293237

[25] Sun D Feng L Shi H Chen J Cui X Chen H . Identification of two novel B cell epitopes on porcine epidemic diarrhea virus spike protein. Vet Microbiol. (2008) 131:73–81. doi: 10.1016/j.vetmic.2008.02.022, 18400422 PMC7117171

[26] Li W Li H Liu Y Pan Y Deng F Song Y . New variants of porcine epidemic diarrhea virus, China, 2011. Emerg Infect Dis. (2012) 18:1350–3. doi: 10.3201/eid1808.120002, 22840964 PMC3414035

[27] Fan B Jiao D Zhang R Zhou J Guo R Yu Z . Origin and epidemic status of porcine epidemic diarrhea virus variants in China. Transbound Emerg Dis. (2020) 67:1364–70. doi: 10.1111/tbed.13444, 31793242

[28] Shi W Hao H Li M Niu J Hu Y Zhao X . Expression and purification of a PEDV-neutralizing antibody and its functional verification. Viruses. (2021) 13:472. doi: 10.3390/v13030472, 33809239 PMC7999980

[29] Wang M Li M Yan G Li H Zhou J Yang A. Epidemiological investigation, isolation, and pathogenicity of porcine epidemic diarrhea virus subtype G2c in Sichuan province. Arch Virol. (2025) 170:129. doi: 10.1007/s00705-025-06308-3, 40377695

[30] Xie B Yan W Yang X Fan H. Molecular characterization of porcine epidemic diarrhea virus in Sichuan from 2023 to 2024. Microb Pathog. (2025) 203:107486. doi: 10.1016/j.micpath.2025.107486, 40097028

[31] Ma X Cui H Huang Y Ma S Chen H. Molecular detection and evolutionary analysis of porcine epidemic diarrhea virus in Henan and Shaanxi provinces in China. Arch Virol. (2025) 170:20. doi: 10.1007/s00705-024-06201-5 39688728

[32] Zhang F Luo Y Lin C Tan M Wan P Xie B . Epidemiological monitoring and genetic variation analysis of pathogens associated with porcine viral diarrhea in southern China from 2021 to 2023. Front Microbiol. (2024) 15:1303915. doi: 10.3389/fmicb.2024.1303915, 38572229 PMC10987963

[33] Zhu P Yuan H Shu X Li X Cui Y Gao L . Epidemiological study and genetic diversity assessment of porcine epidemic diarrhea virus (PEDV) in Yunnan Province, China. Viruses. (2025) 17:264. doi: 10.3390/v17020264, 40007019 PMC11861340

[34] Zhang H Zou C Peng O Ashraf U Xu Q Gong L . Global dynamics of porcine enteric coronavirus PEDV epidemiology, evolution, and transmission. Mol Biol Evol. (2023) 40:msad052. doi: 10.1093/molbev/msad052, 36869744 PMC10027654

[35] Zhu H Feng Z Sun M Zhang S Yang Z Bai J . N-glycosylation of the PEDV spike protein modulates viral replication and pathogenicity. Vet Res. (2025) 56:172. doi: 10.1186/s13567-025-01606-9, 40883772 PMC12395675

[36] Ji Z Shi D Shi H Wang X Chen J Liu J . A porcine epidemic diarrhea virus strain with distinct characteristics of four amino acid insertion in the COE region of spike protein. Vet Microbiol. (2021) 253:108955. doi: 10.1016/j.vetmic.2020.108955, 33373882 PMC7733691

[37] Thavorasak T Chulanetra M Glab-Ampai K Mahasongkram K Sae-Lim N Teeranitayatarn K . Enhancing epitope of PEDV spike protein. Front Microbiol. (2022) 13:933249. doi: 10.3389/fmicb.2022.933249, 35935230 PMC9355140

