# Supplementary figures and images for: Prevalence and S gene characterization of porcine epidemic diarrhea virus in Sichuan province, China (2023–2024)
Source: Front Vet Sci. 2026 Jan 26;12:1748998. doi: 10.3389/fvets.2025.1748998 (PMC12884398; doi:10.3389/fvets.2025.1748998)

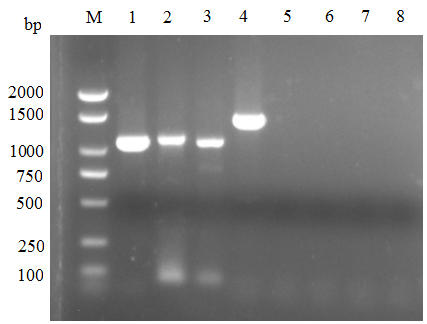

Supplement: Supplementary file 1 [file Image_1.JPEG]
