# Supplementary material for: Prevalence and S gene characterization of porcine epidemic diarrhea virus in Sichuan province, China (2023–2024)
Source: Front Vet Sci. 2026 Jan 26;12:1748998. doi: 10.3389/fvets.2025.1748998 (PMC12884398; doi:10.3389/fvets.2025.1748998)
Supplement: Supplementary file 2 [file Table_1.DOCX]

**Table S1. GenBank accessions for PEDV S gene of reference/detection sequences.**

| Number | Virus strain | Accession no. | Time | Laction | Source |
| --- | --- | --- | --- | --- | --- |
| 1 | CV777 | AF353511.1 | 1978 | Belgium | GenBank |
| 2 | SM98 | GU937797.1 | 1998 | Korea | GenBank |
| 3 | CHM2013 | KM887144.1 | 2013 | China | GenBank |
| 4 | attenuated DR13 | JQ023162.1 | 1999 | Korea | GenBank |
| 5 | AH-M | KJ158152.1 | 2011 | China | GenBank |
| 6 | SD-M | JX560761.1 | 2012 | China | GenBank |
| 7 | AHbz2023-2 | OR808019.1 | 2023 | China | GenBank |
| 8 | FR/001/2014 | KR011756.1 | 2014 | France | GenBank |
| 9 | MYZ-1/JPN/2013 | LC063846.1 | 2013 | Japan | GenBank |
| 10 | OH851 | KJ399978.1 | 2014 | USA | GenBank |
| 11 | USA/Iowa106/2013 | KJ645695.1 | 2013 | USA | GenBank |
| 12 | ZL29 | KU847996.1 | 2015 | China | GenBank |
| 13 | GER/L00719/2014 | LM645058.1 | 2014 | Germany | GenBank |
| 14 | AH2012 | KC210145.1 | 2012 | China | GenBank |
| 15 | CH/JX-2/2013 | KJ526096.1 | 2013 | China | GenBank |
| 16 | IA2 | KF468754.1 | 2013 | USA | GenBank |
| 17 | MN | KF468752.1 | 2013 | USA | GenBank |
| 18 | AH2012/12 | KU646831.1 | 2012 | China | GenBank |
| 19 | AJ1102 | JX188454.1 | 2011 | China | GenBank |
| 20 | CHSD2014 | KX791060.1 | 2014 | China | GenBank |
| 21 | GD-A | JX112709.1 | 2012 | China | GenBank |
| 22 | CH/HNAY/2015 | KR809885.1 | 2015 | China | GenBank |
| 23 | GDS21 | MH726371.1 | 2014 | China | GenBank |
| 24 | SNJ-P | MK702008.1 | 2018 | China | GenBank |
| 25 | BZ2401 | PV609791.1 | 2024 | Bazhong,China | This study |
| 26 | CD2301 | PV609792.1 | 2023 | Chengdu, China | This study |
| 27 | CD2302 | PV609793.1 | 2023 | Chengdu, China | This study |
| 28 | CD2401 | PV609794.1 | 2024 | Chengdu, China | This study |
| 29 | DY2401 | PV609795.1 | 2024 | Deyang, China | This study |
| 30 | DZ2401 | PV609796.1 | 2024 | Dazhou, China | This study |
| 31 | GY2301 | PV609797.1 | 2023 | Guangyuan, China | This study |
| 32 | GY2401 | PV609798.1 | 2024 | Guangyuan, China | This study |
| 33 | LS2401 | PV609799.1 | 2024 | Leshan, China | This study |
| 34 | LZ2401 | PV609800.1 | 2024 | Luzhou, China | This study |
| 35 | LZ2402 | PV609801.1 | 2024 | Luzhou, China | This study |
| 36 | MS2301 | PV614326.1 | 2023 | Meishan, China | This study |
| 37 | MS2302 | PV614327.1 | 2023 | Meishan, China | This study |
| 38 | MY2301 | PV614328.1 | 2023 | Mianyang, China | This study |
| 39 | MY2403 | PV614329.1 | 2024 | Mianyang China | This study |
| 40 | SN2401 | PV614330.1 | 2024 | Suining, China | This study |
| 41 | SN2402 | PV614331.1 | 2024 | Suining, China | This study |
| 42 | SN2403 | PV614332.1 | 2024 | Suining, China | This study |
| 43 | XC2401 | PV614333.1 | 2024 | Xichang, China | This study |
| 44 | ZY2301 | PV614334.1 | 2023 | Ziyang, China | This study |
| 45 | ZY2302 | PV614335.1 | 2023 | Ziyang, China | This study |
| 46 | ZY2401 | PV614336.1 | 2024 | Ziyang, China | This study |
| 47 | MY2302 | PV614337.1 | 2023 | Mianyang, China | This study |
| 48 | ZY2402 | PV641626.1 | 2024 | Ziyang, China | This study |
| 49 | ZG2401 | PV641627.1 | 2024 | Zigong, China | This study |
| 50 | MY2404 | PV641628.1 | 2024 | Mianyang, China | This study |
| 51 | MY2402 | PV641629.1 | 2024 | Mianyang, China | This study |
| 52 | GA2401 | PV641630.1 | 2024 | Gaung'an, China | This study |
| 53 | CD2405 | PV641631.1 | 2024 | Chengdu, China | This study |
| 54 | CD2404 | PV641632.1 | 2024 | Chengdu, China | This study |
| 55 | CD2402 | PV641633.1 | 2024 | Chengdu, China | This study |
| 56 | YA2401 | PV641634.1 | 2024 | Ya'an, China | This study |
| 57 | NC2401 | PV641635.1 | 2024 | Nanchong, China | This study |
