# Supplementary material for: Prevalence and S gene characterization of porcine epidemic diarrhea virus in Sichuan province, China (2023–2024)
Source: Front Vet Sci. 2026 Jan 26;12:1748998. doi: 10.3389/fvets.2025.1748998 (PMC12884398; doi:10.3389/fvets.2025.1748998)
Supplement: Supplementary file 3 [file Table_2.DOCX]

**Table S2. Homology comparison: PEDV strain vs. reference genotypes**

| Virus strain | Percentage of Nucleotide (Amino Acid) Identity (%) | | | | | |
| --- | --- | --- | --- | --- | --- | --- |
|  | CV777 | attenuated DR13 | OH851 | AH2012 | AJ1102 | CHN/HNAY/2015 |
| BZ2401 | 93.8  (92.9) | 93.7  (92.2) | 96.0  (95.1) | 98.1  (97.6) | 97.2  (97.3) | 98.3  (98.0) |
| CD2301 | 93.7  (93.2) | 93.5  (93.0) | 95.9  (95.5) | 97.8  (98.1) | 97.0  (97.7) | 98.4  (98.6) |
| CD2302 | 94.1  (93.6) | 93.8  (93.0) | 96.2  (95.8) | 98.3  (98.3) | 97.5  (98.3) | 98.7  (98.8) |
| CD2401 | 94.0  (92.7) | 93.8  (92.3) | 95.6  (94.3) | 97.8  (96.8) | 97.8  (97.6) | 98.0  (97.2) |
| CD2402 | 94.0  (92.9) | 93.9  (92.5) | 95.7  (94.5) | 97.9  (97.0) | 97.8  (97.8) | 98.1  (97.4) |
| CD2404 | 93.7  (92.5) | 93.5  (91.8) | 94.7  (93.8) | 97.4  (96.8) | 98.8  (98.0) | 97.0  (96.4) |
| CD2405 | 93.7  (93.1) | 93.4  (92.5) | 95.8  (95.4) | 98.0  (98.1) | 97.1  (97.6) | 98.2  (98.2) |
| DY2401 | 94.0  (92.7) | 93.9  (92.2) | 95.7  (94.3) | 97.9  (96.8) | 97.8  (97.6) | 98.1  (97.2) |
| DZ2401 | 94.7  (94.6) | 94.8  (94.3) | 97.3  (97.1) | 95.7  (95.3) | 94.7  (94.7) | 95.6  (95.4) |
| GA2401 | 93.9  (93.5) | 93.7  (93.2) | 96.1  (95.8) | 98.1  (98.3) | 97.3  (98.0) | 98.4  (98.6) |
| GY2301 | 93.8  (93.3) | 93.6  (92.7) | 95.8  (95.4) | 98.1  (97.9) | 97.3  (97.8) | 98.2  (98.3) |
| GY2401 | 94.3  (93.8) | 94.2  (93.3) | 96.2  (95.9) | 99.3  (98.8) | 98.1  (98.5) | 98.5  (98.3) |
| LS2401 | 93.9  (92.4) | 93.9  (92.1) | 95.3  (94.1) | 97.6  (96.9) | 98.1  (97.8) | 97.8  (97.0) |
| LZ2401 | 93.8  (92.6) | 93.6  (92.0) | 94.9  (94.0) | 97.3  (96.9) | 98.7  (98.1) | 97.3  (96.8) |
| LZ2402 | 94.7  (94.6) | 94.9  (94.1) | 97.3  (97.0) | 95.8  (95.1) | 94.7  (94.6) | 95.7  (95.3) |
| MS2301 | 94.0  (93.2) | 93.7  (93.0) | 96.2  (95.7) | 98.1  (98.2) | 97.4  (97.9) | 98.6  (98.7) |
| MS2302 | 93.8  (93.4) | 93.7  (92.9) | 96.1  (95.7) | 98.3  (98.3) | 97.4  (97.8) | 98.6  (98.7) |
| MY2301 | 94.1  (93.5) | 93.9  (93.2) | 96.2  (95.8) | 98.3  (98.3) | 97.4  (98.1) | 98.6  (98.8) |
| MY2302 | 94.0  (93.5) | 93.7  (92.8) | 96.2  (95.7) | 98.2  (98.3) | 97.3  (97.9) | 98.6  (98.6) |
| MY2402 | 93.8  (92.8) | 93.6  (92.3) | 95.9  (95.0) | 97.9  (97.4) | 97.0  (97.1) | 98.1  (97.8) |
| MY2403 | 94.6  (94.1) | 94.9  (93.9) | 97.3  (96.6) | 95.8  (94.9) | 94.9  (94.4) | 95.8  (94.9) |
| MY2404 | 93.8  (92.8) | 93.8  (92.4) | 96.0  (95.0) | 98.3  (97.8) | 97.4  (97.3) | 98.4  (97.8) |
| NC2401 | 94.0  (92.8) | 93.9  (92.5) | 95.7  (94.4) | 98.0  (97.0) | 97.9  (97.8) | 98.2  (97.3) |
| SN2401 | 93.8  (92.6) | 93.6  (92.0) | 94.8  (94.0) | 97.5  (97.0) | 98.9  (98.2) | 97.1  (96.6) |
| SN2402 | 94.8  (94.9) | 95.0  (94.5) | 97.4  (97.3) | 95.9  (95.5) | 94.9  (94.9) | 95.8  (95.7) |
| SN2403 | 94.6  (94.1) | 94.9  (93.8) | 97.0  (96.6) | 95.5  (94.7) | 95.1  (94.7) | 95.5  (94.7) |
| XC2401 | 93.8  (92.4) | 93.8  (92.1) | 95.3  (94.1) | 97.6  (96.9) | 98.1  (98.0) | 97.8  (97.0) |
| YA2401 | 94.0  (92.8) | 93.8  (92.3) | 95.6  (94.3) | 97.9  (96.9) | 97.8  (97.7) | 98.1  (97.3) |
| ZG2401 | 94.0  (92.8) | 93.9  (92.5) | 96.1  (95.1) | 98.1  (97.6) | 97.1  (97.0) | 98.2  (97.8) |
| ZY2301 | 93.8  (93.4) | 93.5  (93.0) | 96.0  (95.7) | 98.0  (98.4) | 97.2  (98.1) | 98.5  (98.8) |
| ZY2302 | 94.1  (93.7) | 93.9  (93.3) | 96.2  (95.9) | 98.3  (98.6) | 97.4  (98.1) | 98.5  (98.8) |
| ZY2401 | 93.7  (93.1) | 93.5  (92.6) | 95.8  (95.4) | 98.0  (98.1) | 97.1  (97.7) | 98.2  (98.3) |
| ZY2402 | 93.7  (92.8) | 93.6  (92.2) | 95.0  (94.1) | 97.6  (97.0) | 98.6  (98.1) | 97.2  (96.7) |
